# Experience of a telehealth and education program with maternal and perinatal outcomes in a low-resource region in Colombia

**DOI:** 10.1186/s12884-022-04935-1

**Published:** 2022-07-29

**Authors:** María Fernanda Escobar, María Paula Echavarria, Hilda Vasquez, Daniela Nasner, Isabella Ramos, María Antonia Hincapié, Stephanie Pabon, Juan Pedro Kusanovic, Diana Marcela Martínez-Ruíz, Javier Andrés Carvajal

**Affiliations:** 1 High Complexity Obstetric Unit, Department of Obstetrics and Gynecology, Fundación Valle del Lili, Cra 98 Nro.18-49, Cali, 760032 Colombia; 2 Department of Obstetrics and Gynecology, School of Medicine, Universidad Icesi, Cali, Colombia; 3 Department of Telemedicine, Fundación Valle del Lili, Cra 98 Nro.18-49, Cali, 760032 Colombia; 4 Centro de Investigaciones Clínicas, Fundación Valle del Lili, Cra 98 Nro.18-49, Cali, 760032 Colombia; 5 Department of Obstetrics and Gynecology, Center for Research and Innovation in Maternal-Fetal Medicine (CIMAF), Hospital Sótero del Río, Santiago, Chile; 6 Division of Obstetrics and Gynecology, School of Medicine, Pontificia Universidad Católica de Chile, Santiago, Chile

**Keywords:** eHealth, Maternal morbidity, Maternal mortality, Eclampsia, Transfusion, Perinatal death

## Abstract

**Introduction:**

Maternal morbidity and mortality rates associated with perinatal care remain a significant public health concern. Rural populations from low and middle-income countries have multiple barriers to access that contribute to a lack of adherence to prenatal care, and high rates of maternal mortality and morbidity. An intervention model based on telehealth and education was implemented between a tertiary high complex care hospital and a second-level hospital from a limited source region.

**Objectives:**

We sought to identify an association in maternal and perinatal care quality indicators after implementing a model based on telehealth and education for patients with obstetric emergencies between two hospitals in a southwestern region of Colombia.

**Methods:**

We conducted an ecological study between 2017 and 2019 to compare before and after obstetric emergency care through telemedicine from a secondary care center (Hospital Francisco de Paula Santander-HFPS) to the referral center (Fundación Valle del Lili-FVL). The intervention included verification visits to determine the installed capacity of care, a concerted improvement plan, and on-site educational training modules in obstetric and perinatal care.

**Results:**

There were 102 and 148 patients treated before and after telemedicine implementation respectively. Clinical indicators after model implementation showed a reduction in perinatal mortality of 29%. In addition, a reduction in the need for transfusion of blood products due to postpartum hemorrhage was observed as well as the rate of eclampsia.

**Conclusions:**

Implementing a model based on telehealth and education between secondary and tertiary care centers allowed the strengthening of the security of care in obstetric emergencies and had a positive effect on perinatal mortality.

**Supplementary Information:**

The online version contains supplementary material available at 10.1186/s12884-022-04935-1.

## Introduction

Rural populations are commonly characterized by poverty, low educational level, and poor access to health services as well as a lack of adherence to prenatal care, which is frequent in the obstetric population. For the year 2021, 2 years after the start of the COVID 19 pandemic, the maternal mortality ratio (MMR) in Colombia was estimated at 78,3 deaths per 100,000 live births (LB) with an important increase in nine territorial entities where the MMR reached more than 100 deaths per 100,000 representing a significant increase in MMR over previous years [1, 2].

According to the third Sustainable Development Goal (SDG), by the year 2030, the MMR should be estimated at less than 70 cases per 100,000 live births, and no country should have an MMR that exceeds twice the world average [3]. In 2017 an MMR of 211 per 100,000 LB was reported globally and it was estimated that between 50 and 100 women experienced near-miss mortality (NMM) for each maternal death [4]. Nevertheless, the novel coronavirus pandemic has had a global impact on maternal and perinatal health via an increase in the MMR, perinatal mortality rate, near-miss mortality, and neonatal morbidity, mainly affecting low and middle-income countries (LMIC) [1, 5].

Most of these deaths in LMIC are preventable and are directly related to the human development index and quality access to obstetric health services [6, 7].

The preceding demonstrates a critical and urgent need to implement innovative and affordable initiatives to improve maternal and perinatal health indicators [8, 9].

Digital health or eHealth is defined as information and communication technology (ICT) in health services, and the surveillance of diseases of public health interest. The implementation of eHealth has technological, cultural, and financial barriers associated with transmitting video, audio, and images in LMIC [8]. The Resolution of the World Health Assembly on Digital Health, recognized the value of digital technologies in contributing to the achievement of the SDGs, establishing the use of telemedicine between trained and certified providers (hospitals) as one of the strategies with the most significant impact [9]. Access to a low number of qualified health workers, geographic inaccessibility, and an unequal distribution of workers contribute to limitations in the adequate coverage of human resources for health barriers intended to be overcome by telemedicine.

In 2018 an emergent intervention model based on telehealth and education was implemented between two hospitals: Fundación Valle del Lili (FVL) a tertiary care medical center from Cali-Colombia, one of the main economic and industrial centers of the country with the only High Complex Obstetric Unit (HCOU) in the region, and Hospital Francisco de Paula Santander (HFPS) a second-level hospital located in a small, low resource region with scarce access to health services and affected by poverty, armed conflict and low educational levels in the municipality of Santander de Quilichao in the northern region of the Cauca department, which corresponds to one of the poorest regions in the country [10, 11].

We aimed to describe an association between the implementation of telehealth and educational model and maternal and perinatal outcomes of patients treated jointly for obstetric emergencies between these two hospitals.

## Methods

### Design and context

We conducted a descriptive ecological study with retrospective information collection aiming to compare maternal and perinatal outcomes before and after the implementation of a telemedicine and educational intervention addressed to support the management of obstetric patients going through an obstetric emergency between two hospitals in the southwestern region of Colombia. The study period before implementation was defined as “Period 1”, established between March 1st, 2017, and July 31st, 2018; while “Period 2” was established between August 1st, 2018, and December 31st, 2019, covering the period during which implementation was carried out.

The FVL Institutional Review Board approved the study protocol on January 22, 2020 (approval number 1560). FVL is a nonprofit highly complex university hospital and is a referral medical care center for patients from the southwestern region of Colombia. FVL is approximately 46 km away from HFPS, which is a secondary level hospital equipped for attending patients with medium complexity diseases from the urban, rural, and dispersed rural areas of 14 municipalities. However, Santander de Quilichao has an MMR of 129 deaths per 100,000 LB according to the official statistics reported by the Ministry of Health and Social Protection (MHSP), which is almost double the MMR of the country in the same period (65 per 100,000 LB - National Health Institute). Furthermore, the near-miss mortality ratio (NMMR) of the territorial entity to which the hospital belongs is also higher than the average reported in Colombia (52 per 1000 LB vs. 38 per 1000 LB) [10, 12].

### Overview of the telehealth- educational model

The institutional factors that directly impact the MMR and preventable NMMR include the availability of qualified human resources and the logistic conditions of care that allow complete and adequate management [13–15].

The implementation of the model began with visits by the FVL medical group to determine the installed capacity of HFPS for obstetric emergencies and the adoption of a telemedicine service. Both institutions conducted a concerted improvement plan based on quality policies arranged for Colombia’s health care processes [16].

Educational modules in obstetric emergencies were conducted in HFPS, including on-site educational workshops supported by simulation activities and interactive lectures that were 6 to 12 hours long. Modules were mainly directed to encourage prompt identification of obstetric emergencies, management of the safe delivery of care, postpartum hemorrhage, hypertensive disorders of pregnancy, maternal sepsis, maternal cardiorespiratory arrest, and early neonatal resuscitation. Emphasis was made on the use of the Modified Early Obstetric Warning System (MEOWS) to detect clinical signs of deterioration in pregnant women with critical illnesses [17]. Modules were addressed encouraging changes in the behavior of the teams in the presence of critical events in pregnant women and the adoption of telehealth strategies.

The group of participants included nursing assistants, registered nurses, general practitioners, and gynecologists, trainers who were gynecologists specialized in intensive care with experience in face-to-face and virtual educational processes, and neonatologists from FVL, who also offered continuous support via chat using the WhatsApp platform with HFPS health staff, which allowed us to emphasize the use of communication strategies and strengthen nontechnical skills [17].

To estimate any changes in knowledge, participants were evaluated before and after the training module. A total of three retraining workshops and four follow-up visits were held during the intervention and trainers organized group-specific teleconferences at least once every 3 months to discuss implementation, compliance, or difficulties.

Liliconnect a platform developed by FVL was enabled to legally exchange information and to record the clinical history of each patient for both institutions, and licensed communication platforms were used for telehealth. At the beginning of the intervention, WebEx was the platform used for this purpose, and Microsoft Teams was later adopted. Telecalls were attended by a nursing assistant who transferred the call to the obstetrician-gynecologist specialized in intensive care, being the way to guarantee and provide coverage 24 hours a day, every day of the week.

To provide care, patients signed a digitized informed consent form; the costs derived from this process were assumed by the health system as established by Colombian regulations. The transfer of patients to the corresponding level of complexity for care was conducted according to their clinical condition (urgent or emergent) and was covered by health insurance, and the government for this purpose.

### Population

The inclusion criteria were pregnant women with obstetric emergencies referred from HFPS for management at FVL for periods 1 and 2. Period 2 had the added criteria of being attended at HFPS for an obstetric emergency and being seen through the telemedicine service, with further referral to FVL after the telecall. For both periods, the exclusion criteria were obstetric emergencies at HFPS referred to other institutions (28 patients), and maternal near-miss criteria were defined according to the guidelines of the Colombian Ministry of Health [18].

A total of 250 patients with obstetric emergencies from the HFPS were included in this study, 102 before and 148 after the implementation of the model based on telehealth and education. Data were retrospectively collected from medical records [18].

### Variables

Information on the sociodemographic and clinical characteristics was collected, as well as maternal and perinatal outcomes. The variables were defined as follows:

- Postpartum hemorrhage: Cumulative blood loss of greater than or equal to 500 mL during vaginal delivery or 1000 mL or blood loss accompanied by signs or symptoms of hemodynamic instability [19].
- Major surgery: Procedures other than childbirth or cesarean section for the management of an obstetric complication or any condition generated because of a serious complication of the woman.
- Eclampsia: New onset of seizures or coma in a pregnant woman with preeclampsia [20].
- Hypertensive crisis: Persistent (lasting 15 min or more), acute-onset, severe hypertension, defined as systolic BP greater than 160 mmHg or diastolic BP > 110 mmHg in the setting of preeclampsia or eclampsia [21].
- Sepsis: A life-threatening condition defined as organ dysfunction caused by an infection during pregnancy, delivery, puerperium, or after an abortion [22].
- Maternal near-miss mortality: When a woman nearly dies but survives a complication during pregnancy, childbirth, or within 42 days of the termination of pregnancy. The criteria were defined according to the guidelines of the Colombian Ministry of Health [23].
- Maternal mortality: Female deaths from any cause related to or aggravated by the pregnancy or its management (excluding accidental or incidental causes) during pregnancy or childbirth or within 42 days of the termination of pregnancy, irrespective of the duration and site of the pregnancy [4].
- Perinatal mortality: The number of fetal deaths past 22 completed weeks of pregnancy plus the number of deaths among live-born children up to 7 completed days of life per 1000 total births (live births and stillbirths) [24, 25].
- Antepartum mortality: Fetal death occurring before labor and/or birth [26].
- Intrapartum mortality: Neonatal death that occurs during labor and birth [26].
- Postpartum mortality: Death of a live newborn within 1–28 days after birth, which is divided into three categories (very early neonatal death, early neonatal death, and late neonatal death) [26].
- MEOWS at admission: Modified Early Obstetric Warning System composed of physiological parameters with a predetermined threshold that determines evaluation, treatment, or intervention [17, 27].
- Medical conditions: Chronic conditions, such as hypertension, preexisting diabetes mellitus, rheumatologic diseases, renal disease, etc. [28, 29].

### Statistical analysis

The unit of analysis was HFPS. The normality of the data was tested using the Shapiro-Wilk and Kolmogorov-Smirnov tests. A descriptive analysis of the variables was expressed by percentages and absolute frequencies for qualitative variables, means, and standard deviations for quantitative variables with normal distributions and medians and interquartile ranges (IQRs) for those not normally distributed. The Mann-Whitney U test and the chi-square or Fisher’s exact tests were conducted to compare sociodemographic, pregnancy, and clinical characteristics between the two periods. A *p* value of < 0·05 was considered statistically significant. Multiple logistic regression estimated the effect of the telemedicine program on the maternal outcome. This variable indicated if a woman’s pregnancy presented PPH, the need for blood or blood components, the need for significant surgery for PPH, eclampsia, hypertensive crisis, or maternal near-miss. A multivariate skewed logistic regression estimated the OR-adjusted telemedicine program on perinatal mortality. For both regressions, the covariables were type of insurance, area of origin, occupation, parity (< 3 or > 3), and medical conditions; specifically for perinatal mortality, the MEOWS at admission FVL, vaginal delivery, cesarean section, and maternal outcome were added. The selection of variables was made with the backward method. We adjusted *p* values for multiplicity with the false discovery rate (FDR) method. The statistical package used was Stata v.14 (StataCorp LLC, College Station, Texas, USA).

## Results

Comparisons of sociodemographic, pregnancy, and clinical characteristics between the two periods are shown in Table 1. The median maternal age was 24 years (IQR: 20–29) for Period 1 and 25 years (IQR: 21–30) for Period 2. In Period 1 there was a high proportion of women belonging to the government health insurance, 68% (69) vs. 50% (74) in Period 2, women frequently had other health insurance in Period 2. This study found a higher frequency of women with technical or college degrees in Period 2 than in Period 1. These reported differences were statistically significant. The differences in proportions of gravidity, parity and gestational age at admission were not statistically significant. Approximately 40% of patients in both groups had a chronic medical condition. There were no differences in the MEOWS score at admission or in the delivery route among patients referred to FVL. There was a significant difference in the number of patients with PROM and preterm labor in Period 2.

**Table 1:** Comparison between the implementation periods regarding the sociodemographic, pregnancy, and clinical characteristics of the care of patients in obstetric emergencies

Characteristics	Period 1, ***N*** = 102 N (%)	Period 2, ***N*** = 148 N (%)	***p***-value
**Age**	24 (20–29)	25 (21–30)	0.43
**Health insurance**			
Subsidized	69 (67.6)	74 (50)	0.006
Contributive	26 (25.5)	37 (25)	0.93
Another regimen	7 (6.9)	37 (25.0)	0.0002
**Area of origin**			
Rural	30 (29.4)	56 (37.8)	0.17
**Occupation**			
Employed/Independent	13 (18.1)	15 (13.8)	0.43
Housewife/ Unemployed	59 (81.9)	94 (86.2)	0.43
**Level of education**			
Primary	18 (23.4)	21 (18.1)	0.37
High School	48 (62.3)	65 (56.1)	0.38
Technical or college degree	11 (14.3)	30 (25.9)	0.06
**Gravidity**			
1	48 (47.1)	52 (35.1)	0.06
2	30 (29.4)	54 (36.5)	0.24
3	12 (11.7)	21 (14.2)	0.58
≥ 4	12 (11.7)	21 (14.2)	0.58
**Parity**			
0	45 (44.1)	54 (36.5)	0.22
1	31 (30.4)	50 (33.8)	0.57
2	13 (12.7)	19 (12.8)	0.98
≥ 3	13 (12.7)	25 (16.9)	0.37
**Gestational age at admission**	35 (32–37)	35 (32–36)	0.47
**Clinical characteristics**			
**Maternal medical conditions**	42 (41.2)	59 (39.9)	0.835
**Preterm labor/ PPROM**	30 (29.4)	66 (44.6)	0.015
**MEOWS at admission to FVL**			
Score 0	18 (17.6)	32 (21.6)	0.44
Score 1–3	68 (66.7)	103 (69.6)	0.62
Score 4–5	9 (8.8)	6 (4.1)	0.12
Score ≥ 6	7 (6.9)	7 (4.7)	0.47
**Termination of pregnancy**			
Vaginal delivery	42 (41.2)	65 (43.9)	0.67
Cesarean section	43 (42.2)	55 (37.2)	0.43
Miscarriage	3 (2.9)	2 (1.3)	0.38
Remain Undelivered	12 (11.8)	26 (17.6)	0.21

The results are expressed as the median (IQR) or number (%).

*MEOWS* Modified Early Obstetric Warning Score

Table 2 shows the distributions between both periods of the maternal clinical outcomes. There were differences in the need for blood product transfusion, which was lower in the group of pregnant women managed after the model implementation (12 [11·7%] vs. 7 [4·7%]) and this group also showed a reduction in the proportion of obstetric patients with eclampsia (7 [6·8%] vs. 2 [1·3%]). The near-miss maternal mortality was similar between the two groups. There were no maternal deaths in either period. Differences in the proportion of perinatal deaths (13 [12·7%] vs. 4 [2·7%]) were observed after the implementation of the model based on telehealth and education, with a greater magnitude of perinatal mortality in the postpartum period mainly due to extreme preterm deliveries. During Period 1, 98% (100/102) of the patients were admitted to the HCOU, of whom 43% (44) required ICU management. There were no differences compared to Period 2, since 98% (145/148) were admitted to the HCOU, and 34% (51) required management in the ICU. There were also no differences in birth weight, admission to the neonatal ICU, and ICU length of stay.

**Table 2:** Description of maternal and perinatal indicators of the care of patients referred from HFPS to FVL in obstetric emergencies by implementation period

Characteristics	Period 1, ***N*** = 102 N (%)	Period 2, ***N*** = 148 N (%)
**Adverse Maternal outcomes**	52 (51.0)	70 (47.3)
ICU admission	44 (43.1)	51 (34.5)
ICU/Obstetric ICU length of stay	2 (2–4)	2 (1–4)
HCOU admission	100 (98.0)	145 (97.9)
HCOU length of stay	3 (2–4)	3 (2–4)
PPH	11 (10.8)	13 (8.8)
Need for blood and blood components	12 (11.8)	7 (4.7)
Need of major surgery for PPH	4 (3.9)	1 (0.7)
Eclampsia	7 (6.9)	2 (1.3)
Hypertensive crisis	23 (22.5)	29 (19.6)
Maternal Near Miss mortality	41 (40.2)	66 (44.6)
**Perinatal outcomes**		
Newborn weight		
< 2500 g	67 (65.7)	104 (70.3)
> 2500 g	35 (34.3)	44 (29.7)
NICU admission	49 (48.0)	75 (50.7)
NICU length of stay	5 (3–13)	6 (4–9)
**Perinatal mortality**	13 (12.7)	4 (2.7)
Antepartum	4 (30.8)	1 (25.0)
Intrapartum	4 (30.8)	0 (0)
Postpartum	5 (38.5)	3 (75.0)

The results are expressed as the median (IQR) or number (%)

*PPH* Postpartum hemorrhage, *PPROM* Preterm prelabour rupture of membranes, *ICU* Intensive Care Unit, *HCOU* High Complexity Obstetric Unit, *NICU* Neonatal Intensive Care Unit

We found a 95% confidence interval in which the odds adjusted for maternal outcomes spanned from a reduction of 50% to a considerable increase of 40% after implementing the telemedicine program; suggesting no significant statistical evidence of differences between the two periods. Conversely, we observed a significant decrease of 78% in perinatal mortality; however, with 95% confidence, this reduction can be 29% or 97% (Table 3).

**Table 3:** Effect of the telemedicine program implementation on maternal outcomes and perinatal mortality

Outcome	OR crude (IC95%) ***p***-value	OR adjusted (IC95%) ***p***-value adjusted for FDR
Maternal outcomes	0.9 (0.5–1.5); p = 0.56	0.85^a^ (0.5–1.4); *p* = 0.56
Perinatal mortality	0.19 (0.04–0.64); 0.002	0.22^b^ (0.07–0.71); *p* = 0.022

^a^OR Adjusted for Health insurance and level of education. ^b^ OR adjusted for education level, parity> 3, cesarean section, pregnancy outcomes, MEOWS at admission to FVL

## Discussion

### Principal findings

This study showed that a model based on telehealth and education for the care of obstetric emergencies between two hospitals of medium and high complexity in southwestern Colombia significantly reduces perinatal mortality. The strategy allowed health professionals to optimally direct the management of patients and reduce transfer times to a higher level of complexity, which may be related to these results. An effect statistically significant for maternal outcome was not found, however, a reduction in the need for transfusion of blood products due to postpartum hemorrhage (PPH), as well as the rate of eclampsia was found.

The reduction in perinatal mortality found was an encouraging result for the teams from both institutions and, according to the evidence available in LMIC, can also be the result of strengthening the competencies of health personnel [30–32]. However, the characteristics of the intervened population in Period 2, were different in terms of rurality, with the educational level to changing the found effect.

The institutional factors that directly affect the MMR and the preventable NMMR in up to 90% of the events include the availability of qualified human resources and the logistic conditions of care that allow complete and adequate management [13, 14]. The use of telemedicine between highly complex hospitals and rural areas lacking specialists and environments with limited infrastructure can improve diagnosis, management, and patient outcomes [8, 15]. The adoption depends on the acceptability of equipment, effectiveness, feasibility, use of resources, and indications for equity, gender, and rights.

Two meta-analyses in resource-limited settings showed that basic neonatal resuscitation decreased birth-related deaths (RR 0.70, 95% CI 0.59 to 0.84) [33] and that basic training in neonatal resuscitation decreased the incidence of stillbirth (RR 0.79, 95% CI 0.44 to 1.41), mortality in the first 7 days (RR 0.53, 95% CI 0 .38 to 0.73), neonatal mortality at 28 days (RR 0.50, 95% CI 0.37 to 0.68) and perinatal mortality (RR 0.63, 95% CI 0.42 to 0.94) [34]. This impact includes delivery care by trained personnel and a reduction in perinatal mortality (RR 0.77, 95% CI: 0.69 to 0.85) [35]. In our model based on telehealth and education, knowledge of delivery care, monitoring for the detection of cases with nonreassuring fetal states, timely referral of pregnant women at high risk for perinatal mortality, and the standardization of fetal resuscitation in utero, and neonatal resuscitation processes were strengthened.

Although the differences did not reach significance, we consider it essential to highlight the reduction in transfusions and eclampsia events in patients referred to FVL after implementing our model. In the case of PPH, medical treatment in the first stages using intervention packages can determine efficient care without transfusions and surgical interventions. For this reason, the reduction of transfusion requirements may be an indirect marker of the improvement of care in the medium complexity hospital where the bleeding event occurred, especially when the availability of products for transfusion is insufficient for all LMIC needs [36].

Eclampsia is also a clinical entity sensitive to the quality of care provided during preeclampsia. The disparity in the incidence of eclampsia between ICH and LMICs is related to time management and the availability of resources for care [20]. Standardized protocols to prevent eclampsia determine that eclamptic seizures occur in less than 0 6% of pregnant women who receive magnesium sulfate [37]. Additionally, early management of severe hypertension decreases the risk of cerebrovascular accidents and eclampsia [38], if medications are administered within 30 to 60 minutes after the hypertensive emergency diagnosis [39]. These principles were used in the educational process by creating mental maps shared between the teams of both hospitals and implemented in the management evaluated by telemedicine for preeclampsia cases, which were probably responsible for decreasing the proportion of patients with eclampsia.

The implementation of a telemedicine service among health providers in a middle-income country aims to overcome the historical barriers defined for this type of service [40] and demonstrate the effectiveness, viability, use of resources, and implications for equity, gender, and rights that have been established by the WHO [41].

Tele-emergency services have been considered a potentially life-saving technology, allowing the expansion of the obstetric team during critical events, shortening the time of care, improving coordination and promoting patient-centered care. However, one of the problems that makes the use more difficult is the technology adoption process, especially in low-resource settings with low exposure to technology, for this reason, implementing a support and educational model based on the needs of the less complex hospital was a definitive process for the technology adoption and the final use of the telemedicine service. The results of this project can generate an option for innovation in obstetric care by telehealth, promoting the reporting of similar strategies at a global level. The telehealth application in obstetrics includes prenatal control and sexual and reproductive health, but the care of patients in obstetric emergency settings has not been reported [42].

### Limitations and strengths

Our study has the weaknesses inherent to the “before and after” evaluations, especially the loss of information in the period before implementation, where there were no records of pregnant women in obstetric emergencies treated at HFPS and who were not referred to FVL. The main challenges for adopting a telemedicine program include the administrative commitment of health institutions and insurers, cost-effective infrastructure, and, ultimately, sustainability [8]. In our case, all the challenges described were presented during the internal structuring process of the telemedicine program. The institutional operational infrastructure was carried out during the 9 months before the start of the teleconsultations. However, the development of solid strategic alliances between both hospitals and the commitment of the health secretaries of Santander de Quilichao and the Cauca Department allowed the consolidation of this strategy.

In Period 1, the education and preparation processes designed between both institutions were essential for the project’s success. Implementing intervention packages with the development of checklists, using modified early warning systems in obstetrics, procedural documentation templates, and simulation educational modules with a demonstrated impact on the management of obstetric emergencies [42, 43]; these strategies were implemented in FVL in 2017, and adapted for replication in this project. Nevertheless, the experience has shown that change in the actions of medical teams using all these inputs are only possible if they incorporate the concepts of communication, teamwork, and safety culture, even in telehealth processes [14, 43–45]. All these aspects were evaluated for both institutions and permanent monitoring from a more complex level allowed hospital teams of medium complexity to understand and incorporate these concepts, recover security and reliability in the health system and gain adherence to the project, especially when the team was recognized for the good results achieved.

No other models that adopt telehealth and collaborative educational strategies have been proposed in Latin America. Our study promotes the adoption of this type of strategy in developing countries, evidencing that this type of intervention positively impacts the burden of disease represented by obstetric emergencies.

### Future implications

There is a regionalization model for obstetric care by levels in Colombia, concentrating very high-risk patients in very high-complexity centers, according to the evidence. In 2015, the American College of Obstetricians and Gynecologists (ACOG) and the Society of Maternal-Fetal Medicine established levels of maternal care [46], considering that obstetric complications increase in hospitals with a low volume of deliveries and high-risk patients and that almost 59% of births occur in hospitals with less than 1000 deliveries per year where obstetric emergency events may occur. Therefore, efforts must be made in all institutions regardless of the level or volume of care.

Most LMICs do not have many highly trained medical staff members to manage obstetric emergencies and critical obstetric care, these staff members are concentrated in the most complex hospitals [47]. The possibility of connecting the medical staff of low and high-complexity hospitals optimizes human resources and increases the chances of better maternal and perinatal outcomes. The experience of implementing the model can give impetus to the best use of telehealth strategies. For future projects, it is essential to establish the qualitative measurement of the impact that this program has on every team, which would help support the results of the implementation of technology between two health institutions.

## Conclusion

Strengthening the technical and nontechnical competencies of the medical team at the lowest complexity level with a standardized education system, the implementation of a structured telemedicine service, the shared use of protocols and intervention packages between institutions, and associations among all the actors in the health system, allowed the development of a model of care for obstetric emergencies with an impact on maternal and perinatal indicators. Despite the advantages that telemedicine offers, health systems face multiple barriers to effective implementation. There remains a large gap in the integration of this type of proposal into the daily performance of obstetricians/gynecologists around the world, especially in LMICs.

## Supplementary Information

**Additional file 1: Supplementary file 1.** Research in context.

## Data Availability

The datasets analyzed during the current study and that support the findings of this study are available from Fundación Valle del Lili (FVL), but restrictions apply to the availability of these data, due to internal privacy policies. Data are however available from Martinez-Ruiz D. M upon reasonable requests and with permission of Fundación Valle del Lili.
