# Supplementary material for: Experience of a telehealth and education program with maternal and perinatal outcomes in a low-resource region in Colombia
Source: BMC Pregnancy Childbirth. 2022 Jul 29;22:604. doi: 10.1186/s12884-022-04935-1 (PMC9336139; doi:10.1186/s12884-022-04935-1)
Supplement: Supplementary file 1 — Additional file 1: Supplementary file 1. Research in context. [file 12884_2022_4935_MOESM1_ESM.docx]

**Supplementary file 1:** Research in context.

**Research in context box**

**Evidence before this study**:

Tele-emergency services have been regarded as potentially life-saving technology by providing immediate and synchronous audio/video connections, commonly between low-volume rural hospitals and an urban emergency department. Its implementation allows the expansion of the care team during critical events, shortens care time, improves care coordination, and promotes patient-centered care. Medium-quality evidence suggests that the use of digital health in rural areas lacking specialists and settings with limited infrastructure can improve the diagnosis, management, and outcomes of patients from multiple specialties, with weak evidence for the integration of telehealth in maternal care. A systematic review of 47 articles that included obstetrics, family planning, and gynecology results suggested benefits with text messages and remote monitoring for the decrease in the number of unscheduled visits. There are no published reports for the care of patients with high obstetric risk or in obstetric emergency settings.

**Added value of this study:** Telemedicine among hospitals of different complexity levels for the management and referral in better conditions and with fewer delays can generate an option for innovation in obstetric care by telehealth, promoting the reporting of similar strategies globally. Maternal mortality reduction projects supported by innovation processes are necessary after reports of deterioration in all human development indicators globally.

**Implications of all the available evidence:**

The strengthening of the technical and non-technical competencies of the medical team at the least complex level with a standardized education system, the implementation of a structured telemedicine service, the shared use of protocols and intervention packages between institutions, and alliances between All the actors of the health system allowed the development of an effective care model for the care of obstetric emergencies with an impact on perinatal health indicators. These models of care can be invaluable amid the COVID-19 pandemic, especially in resource-limited settings with low vaccination rates.

Sup
